# Advances in Elastomers

**DOI:** 10.3390/ma14020348

**Published:** 2021-01-12

**Authors:** Michal Sedlačík

**Affiliations:** 1 Centre of Polymer Systems, Tomas Bata University in Zlín, Tr. T. Bati 5678, 760 01 Zlín, Czech Republic; msedlacik@utb.cz; 2 Department of Production Engineering, Faculty of Technology, Tomas Bata University in Zlín, Vavreckova 275, 760 01 Zlín, Czech Republic

Elastomer materials are characteristic for their high elongation and (entropy) elasticity, which makes them indispensable for widespread applications in various engineering areas, medical applications or consumer goods. Their application-oriented development has to go hand in hand with material resources, economic aspects as well as environmental issues. Not only are elastomer matrix properties of high importance when optimizing the utility properties of the product; the fillers, plasticizers and other compounds are other property-determining factors that need to be considered, as these can positively affect the stimuli-responsive character, novel functionality, biological application, or fracture behavior, among other things, for gaining advances in elastomers [1,2,3,4].

This Special Issue, which consists of seven articles written by research experts in their field of interest, reports on the most recent research on rubber mixtures composition with the emphasis on the final utility properties of the product. Several novel and entrancing methods related to the friction and abrasion, waste management, bio-based composition, as well as two comprehensive reviews on the field-responsive elastomer materials, are introduced.

The environmental issue aspects in terms of hazardous waste management and bio-based resources are the subjects of the first two publications [5,6]. Prochon et al. used buffing dust collagen (BDC) originating from a waste product of the chromium tanning process in the leather industry as a modern filler in styrene butadiene rubber (SBR) [5]. The effect of this biodegradable filler on the physicochemical properties, biodegradation and thermo-oxidative aging of the SBR vulcanizates was investigated. The rod-like shape together with the scleroprotein additives presented on the surface of such a nanofiller led not only to more favorable dispersion within the elastomer, but also increased the cross-link density of the SBR vulcanizates, resulting in enhanced mechanical strength. Another advantage of BDC came from its antioxidant properties stabilizing (through the incorporation of chromium ions) the whole vulcanizate against thermo-oxidative aging, and its intense black color enabling it to be used also as a coloring additive. Finally, the release of compact chromium presented in the BDC filler from the vulcanizate is reduced by the formation of stable interfacial bonds between BDC and SBR.

Continuous material development is essentially important not only in the rubber industry. Rahman et al. proved the advantageous effect of bio-based plasticizers not only from the material resources viewpoint, but also as a processing aid positively affecting the complete process chain [6]. They investigated the suitability of two bio-based plasticizers, namely epoxidized esters of glycerol formal from soybean and canola oil, as sustainable alternatives with lower health risks compared to conventional plasticizers. Acrylonitrile-butadiene rubber with different ratios of monomers was used to observe the compatibility between the NBR and plasticizers for systems with different polarities. The positive effect of the bio-based plasticizers used in this study on the complete process chain was found in the NBR cure-accelerating effect, and the mechanical and thermal properties, which all were better in comparison with the conventional plasticizers-based systems investigated simultaneously.

Pöschl and co-workers followed the trend of eliminating the undesirable mechanical vibrations by vibro-insulating materials [7]. They investigated the ability to damp the mechanical vibration of SBR vulcanizates filled with four types of carbon black. The vibro-insulation tests comprised the forced oscillation method based on the transfer damping function. It was observed that the first resonant frequency decreased with an increase in the carbon black particle size as a higher transformation of input mechanical energy into heat under dynamic loading occurred. Durability experiments on the basis of harmonic loading revealed a shift of the first resonance frequency peak to lower excitation frequencies. The damping of mechanical vibrations had also been correlated with the excitation frequency of the vibration, the sample’s thickness and inertial mass.

Rubber-like materials are frequently working under such conditions as rubbing, abrading, chunking, and tearing, which lead easily to mechanical failure. Vaikuntam et al. contributed with the friction, abrasion, and crack growth behavior of tire tread compounds composed of solution SBR filled with in-situ generated alkoxide silica or commercial precipitated silica [8]. It was demonstrated that a masterbatch containing in-situ silica could be used, with benefits for the industrial production of car tires or technical rubber articles in general, as its vulcanizates possessed a lower friction coefficient in comparison with classical precipitated silica-filled systems, which is promising for the low rolling resistance necessary, for example, for better fuel efficiency. Furthermore, if the samples having similar crosslink densities are compared, a much better resistance to crack growth was again observed for in-situ silica-based system.

For their characteristic properties, elastomer materials are used also in various advanced applications. Smart external field-responsive systems such as these can reversibly change their properties under an external stimulus, represented by an electric or magnetic field, pH, and light, among other things [9]. Aziz and co-workers led the world not only in the characterization of magnetorheological elastomers (MREs) reacting with changes in the systems’ stiffness under the application of an external magnetic field, as is typical for such systems, but also included nanosized Ni–Mg cobalt ferrite particles into these MREs based on conventional carbonyl iron magnetic particles to make them suitable for use as actuators or flexible sensors [10]. Although the demanded electrical properties could be obtained with carbon-based particles, such as graphite or graphene, the necessary high concentration of the filler would negatively increase the stiffness of the system, thus the presented study could solve this problem. The developed MRE alters the magnetic, rheological and electrical resistance behavior appropriately, even at small concentrations (1.0 wt. %) of Ni–Mg nanoparticles.

The development of MREs is described in this Special Issue in two review papers complementing each other [11,12]. A more general review is given by Kang et al., in which the authors concentrate not only on the material basis used in MREs and covering a broad range of polymeric elastomeric materials, including, e.g., waste tire rubber as well, but also on the particles’ distribution within the elastomeric matrix and its effect on the MR performance described in various experimental techniques [11]. Finally, in their review Jamari et al. concentrated on magnetic particles’ coating as an important task necessary in order to obtain a system with improved sedimentation and oxidation stability, and appropriate tribology properties [12]. The chemical methods for particle coating, such as atom transfer radical polymerization, chemical oxidative polymerization or dispersion polymerization, are thoroughly described together with the effect of the chemical nature of the coating on the MR performance.

## Data Availability

Data sharing not applicable.
